# Characteristics and effectiveness of interventions to reduce cyberbullying: a systematic review

**DOI:** 10.3389/fpubh.2023.1219727

**Published:** 2023-08-30

**Authors:** Jesús Henares-Montiel, Guadalupe Pastor-Moreno, Alberto Ramírez-Saiz, Marina Rodríguez-Gómez, Isabel Ruiz-Pérez

**Affiliations:** ^1^Escuela Andaluza de Salud Pública, Granada, Spain; ^2^CIBER en Epidemiología y Salud Pública (CIBERESP), Instituto de Salud Carlos III, Madrid, Spain; ^3^Instituto de Investigación Biosanitaria de Granada (ibs.GRANADA), Granada, Spain; ^4^Servicio de Cardiología, Hospital Clínico Universitario de Valencia, INCLIVA, Valencia, Spain; ^5^Unidad de Hospitalización a Domicilio, Hospital Clínico Universitario de Valencia, Valencia, Spain

**Keywords:** cyberbullying, online bullying, intervention, effectiveness, characteristics, systematic review

## Abstract

**Background:**

This paper presents the results from a systematic review on the effectiveness of interventions to reduce Cyberbullying (CB) as a function of their outcomes and main characteristics; and an analysis of the level of completeness to which the characteristics of these interventions are described.

**Methods:**

Systematic searches were conducted in PubMed, Scopus, ERIC and Psycinfo databases on February 14, 2022. In addition, relevant publications were hand searched for relevant studies. We considered interventions that provided data on CB prevalence changes in populations between primary school and college age.

**Results:**

In total, 111 studies were retained for further screening from 3,477 results. Following rigorous screening, 43 reports including 46 studies and information from 36 different interventions were included in our systematic review. Results shows that most of the interventions measuring reductions in global CB, cyberperpetration/victimization, cybervictimization and cyberperpetration were effective or partially effective. While the interventions measuring reductions in cyber-bystanding were not effective. Multicomponent interventions showed higher effectiveness than single-component interventions. After completion of the TIDieR check-list, included interventions were considered to offer an insufficient level of detail for a number of the analyzed items in relation to “how well planned,” “intervention modifications” and “tailoring.”

**Conclusion:**

Given the aforementioned, it is critical to increase the number of studies and the quality of interventions targeting CB and the level of detail of its description in order to obtain more robust outcomes about how to reduce its prevalence and facilitate the replication of the effective interventions.

**Systematic review registration:**

https://archive.org/details/osf-registrations-wn5u4-v1, Identifer DOI: 10.17605/OSF.IO/WN5U4

## 1. Introduction

Adolescence is an important stage in life. During this life stage, individuals tend to make first contact with information communication technologies (ICTs), with such use then becoming frequent. ICTs have changed the way in which information is created, transmitted and received, not only in the professional area but also in personal and social contexts. This also encompasses different types of violence (1).

Bullying is traditionally considered to consist of a type of aggression that occurs when a young person is exposed, repeatedly or over a long period of time, to negative actions at the hands of other students and in a context in which an imbalance of power exists (2). Although different definitions exist, cyberbullying (CB) is generally understood as a form of bullying that takes place online. In other words, it consists of using new information and communication technologies, mainly the Internet and mobile telephones, to harass and bully peers (3).

CB is a much more recent and unknown phenomenon than bullying. It is often approached from a perspective rooted in what is known about bullying (similarities) but this often fails to consider differences between the two types. The online context of CB creates unique risk, such as anonymity and the speed and spread of CB (4).

Various roles or actors can be identified within the dynamic of CB: victims, aggressors, victimized aggressors and bystanders. Over the past few years, the attention of the scientific community on the so-called cyberbystanders, who are those who witness cyberaggressions, has increased. In general, cyberbystanders may participate in the events by helping or defending the victim and reinforcing the aggressor’s attitude, although they may also act passively, which can be interpreted as an act of approval and may aggravate the situation (5).

With regards to the frequency of CB, results from a recent meta-analysis using data from 25 European Union countries showed prevalences between 2.8–31.5% for cybervictimization (CV), between 3.0–30.6% for cyberperpetration (CP), and between 13.0–53.1% for cyberbystanding (CBS) (6).

The comprehensive review by Zhu et al. (7) evidenced that females were more likely to be cyberbullied than males while, regarding the risk factors of CB perpetration, it is generally believed that older teenagers, especially those aged over 15 years, are at greater risk of becoming CB perpetrators. Although some of these findings are still deemed controversial. In relation to CBS, although males appear to be observers of CB events more frequently (8), females tend to be defenders more frequently than males (9, 10), although in a recent study no such differences were observed (11). No differences by age were found (9). On the other hand, the role of CV-CP is more frequent at older age and in men than in women (12, 13).

The harmful effects of bullying on health have been widely described in various scientific works (14). Given that CB can affect a larger number of individuals, be exercised in an anonymous way and can take place at any time of the day, it has been shown to have more serious and long-lasting consequences for health than traditional bullying. Such consequences range from symptoms of depression, anxiety, low self-esteem, school absenteeism, headaches and physical health issues, to suicidal ideation and/or the act of suicide (15, 16).

Given the increased prevalence of this phenomenon amongst the youth population and the seriousness and longevity of its implications for health, CB has long been identified as a social and public health issue. Given this, various interventions have been elaborated with the main aim of avoiding the emergence of CB and, in this way, trying to decrease its impact on health.

The first articles describing CB interventions appeared in the early 2010s in southern Europe (17, 18), evaluating the effectiveness of these interventions in reducing perpetration and victimization and showing inconclusive results. Soon after, the first studies appeared in central and northern Europe (19, 20) in which a decrease in perpetration figures after intervention was observed. The first studies outside Europe appeared in the United States and Australia in 2015 and 2016, respectively (21, 22). But it is since that date when the publication of articles describing anti-cyberbullying programs and data on their effectiveness has grown the most and references can be found from the Middle East to Latin America (23, 24), as well as in the rest of the regions already mentioned.

Many systematic reviews have been conducted in the recent years to analyze the effectiveness of these interventions, but the current reviews were either outdated and did not include recent studies (25), did not include college-age students (26, 27), were focused on both traditional and CB interventions (25, 28) and/or did not include outcomes regarding CBS behavior (4, 26).

Due to the large proliferation of studies on the development and evaluation of the effectiveness of CB interventions, rather than focusing efforts on developing new intervention programs, it seems reasonable to make an effort to try to synthesize the large amount of available evidence that exists in this regard and to draw conclusions about which of the existing programs are effective and due to what characteristics they are effective or not.

The current study builds on past reviews of the literature to address these issues through the following aims: (1) To identify interventions elaborated to reduce the prevalence of CB in students from primary school to college-age. (2) To analyze the effectiveness of these interventions as a function of their outcomes (CB roles) and main characteristics. (3) To analyze the level of completeness to which the characteristics of these interventions are described in order to enable their effective replication.

## 2. Materials and methods

This study was part of a broader systematic review aiming to identify the prevalence of CB, alongside its associated risk factors, health impact and the effectiveness of interventions to reduce it. The review and its procedures were planned, conducted and reported according to PRISMA 2020 (Preferred Reporting Items for Systematic Reviews and Meta-Analyses) guidelines (29). The review protocol was registered in the Open Science Framework online public database (osf.io/nc4u2).

### 2.1. Information sources and search strategy

We designed specific search strategies for use with the PubMed, Scopus, ERIC and Psycinfo databases. The strategy, which combined MeSH (Medical Subject Headings) terms and keywords, was initially designed for PubMed, and later adapted and used with the other three databases.

We executed the searches on February 14, 2022. No language or date restrictions were applied. A bibliographical database created through Rayyan QCRI (30) was used to store and manage uncovered references. The full search strategy is described in Supplementary Appendix S1.

### 2.2. Study selection

Our inclusion criteria were as follows. First, studies were only included that assessed the effectiveness of interventions aimed at decreasing the prevalence of CB. Second, interventions were required to target students in elementary/primary school, secondary school or college. Finally, studies were required to be randomized controlled trials or quasi-experimental investigations with or without comparison groups.

Pilot studies, studies focusing on outcomes other than the prevalence of CB (such as attitudes towards CB, perceptions of the severity of CB, etc.) and studies written in other language than Spanish or English were excluded.

Two of the reviewers (JHM and ARS) independently screened the titles and abstracts of retrieved documents following implementation of the search strategy in order to ascertain their eligibility. Those meeting inclusion criteria were selected for full text assessment, after which a new independent assessment was performed for final selection of studies for inclusion in the review. Disagreements were resolved through discussion with a third reviewer (IRP). When an intervention was reported in two or more studies, the one that appeared first was taken into account for the qualitative analysis of this RSL.

### 2.3. Risk of bias assessment

We used the Effective Public Health Practice Project (EPHPP) Quality Assessment Tool for Quantitative Studies to assess the risk of bias of included studies (31). Sources of bias related to selection bias, study design, confounders, blinding, data collection methods, and study withdrawal and dropout. Bias was classified into three categories (strong, moderate and weak). Two reviewers (ARS, MRG) independently performed assessments of methodological quality, with a third reviewer (JHM) also being consulted to resolve any disagreements. No articles were excluded based on evaluations of methodological quality.

### 2.4. Data extraction and synthesis of results

A data extraction form was developed for the review and used to collect relevant information from each article, including information about the methods and population characteristics, interventions, comparators, outcomes, timing, settings, and study design. Two independent reviewers (JHM and ARS) extracted the data and outcomes were summarized qualitatively. The qualitative summary included a description of the features and main outcomes of the interventions. We carried out a detailed analysis using the TIDieR (Template for Intervention Description and Replication) framework for describing interventions (32).

Following review of the included articles, we categorized the main outcomes into the following five groups: reduction in global CB, reduction in CP, reduction in CV, reduction in cyberperpetration-victimization (CP-CV) and reduction in CBS.

Intervention strategy components were categorized into three groups in order to operationalize outcome exposure. A strategy was considered to be multi-component when it included two or more of these components. Categories were defined in the following way:

- Educational/Informational: information or educational materials are offered in order to broaden and deepen theoretical knowledge of CB (involved agents, impact on health, etc.)
- Cognitive/Behavioral: strives to impact on the way in which students perceive the phenomenon of cyberbullying and their behaviors in relation to it, generally through activities that promote empathy.
- Skill development: works on developing practical skills which students can put into practice in order to generate safe settings which are free from violence.

We assessed the overall effectiveness of each intervention in order to determine whether significant postintervention improvements were observed in the experimental group relative to the control group. Interventions were determined to be effective when they achieved statistically significant improvements in all of the predetermined groups or at all measured time-points. Partial effectiveness was defined as a statistically significant change in one or more, but not all, of the predetermined groups or measured time-points. Interventions were deemed ineffective if there was no improvement in any of the predetermined groups or at any measured time-point.

### 2.5. Description of interventions

The TIDieR checklist (32) was used to analyze whether included studies reported their experimental interventions in full detail, in terms of “intervention name,” “why” (theoretical framework), “what” (description of the intervention and control), “who” (intervention provider), “how” (use of technology, individual or group sessions), “where” (setting), “when and how much” (duration, number of sessions), “tailoring,” “modifications to the intervention,” “quality of planning” and “quality of implementation” (fidelity/adherence).

## 3. Results

**Figure 1:**
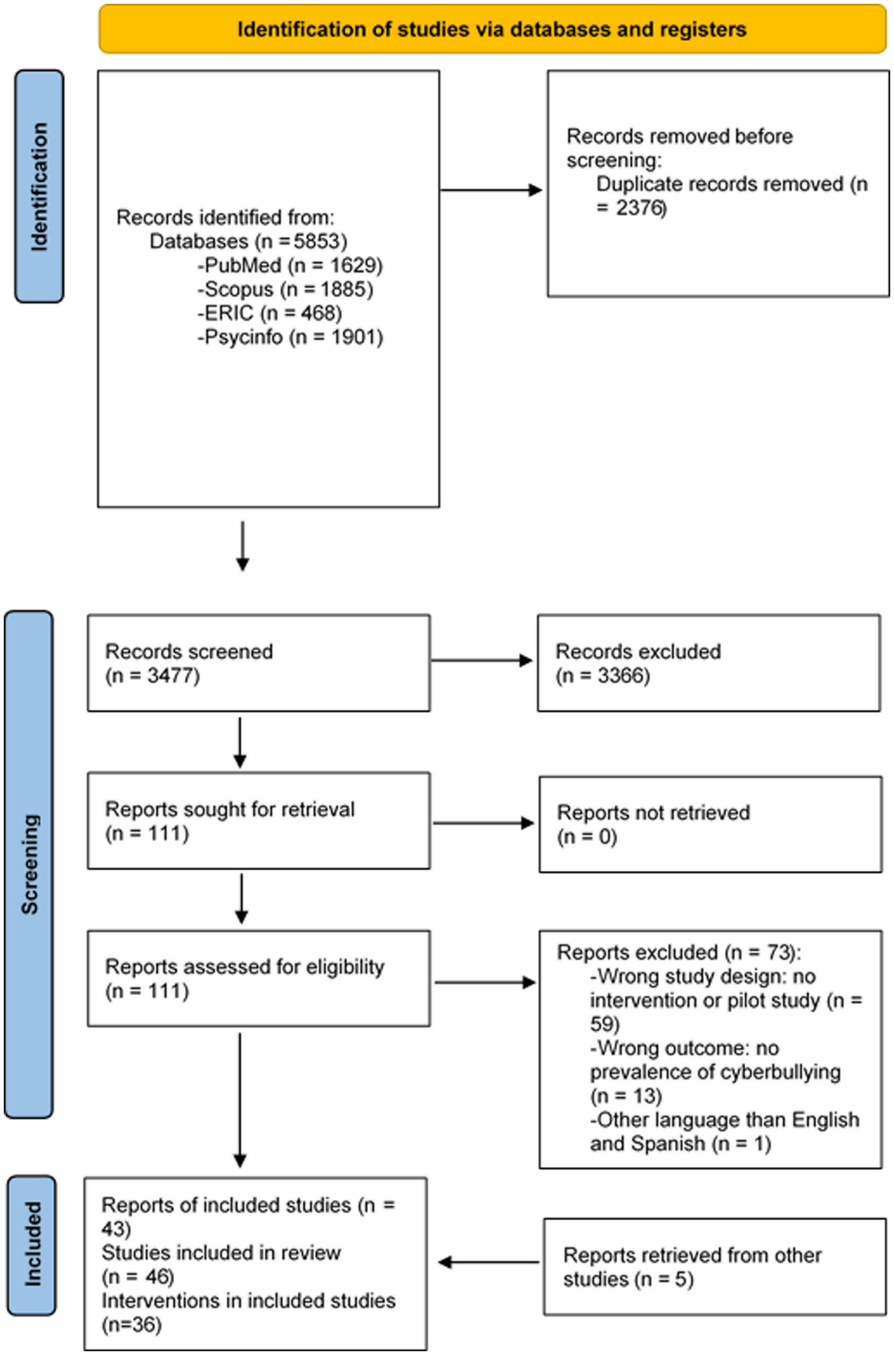
PRISMA 2020 flow-diagram.

Our search results are summarized in a PRISMA flow diagram (Figure 1). A total of 5,853 records were identified in the initial search, 2,376 of which were duplicates. Title and abstract screening of the remaining 3,477 records resulted in the inclusion of 111 citations for further review. Seventy three of these reports were excluded for the following reasons: 59 presented a wrong study design, either they were not intervention studies or they were pilot studies; 13 showed results other than reduction of CB prevalence and another one was written in other language than English or Spanish. Following examination of full-text articles, 38 reports were finally included. Another 5 reports were identified from the reference lists of other publications. Finally, 43 reports were included in the final review with information of 46 studies and 36 different interventions.

### 3.1. Characteristics of the studies

Characteristics of the included studies are summarized in Table 1. Overall, 46 studies were identified. With regards to year of publication, the first studies were published in 2012 and nearly 75% (n = 34) were published after 2015. Twenty-one studies were randomized controlled trials (45.7%), 21 were controlled before-after studies (45.7%) and only 4 were uncontrolled before-after studies (8.6%). Fourteen of these studies were from Spain (30.4%) and 8 were conducted in Italy (17.3%).

**Table 1:** Characteristics of the studies.

Characteristics of studies (*n* = 46)		
*Publication year*	*n*	*%*
2010–2015	12	26.0
2016–2021	34	74.0
*Country*	*n*	*%*
Spain	14	30.4
Italy	8	17.3
US	5	10.9
Germany	4	8.7
Australia	3	6.5
Austria	2	4.3
Israel	2	4.3
Belgium	1	2.2
Cyprus	1	2.2
Finland	1	2.2
Greece	1	2.2
Mexico	1	2.2
Netherlands	1	2.2
Turkey	1	2.2
UK	1	2.2
*Study design*	*n*	*%*
Randomized controlled trial	21	45.7
Controlled before-after study	21	45.7
Uncontrolled before-after study	4	8.6
*Methodological quality*	*n*	*%*
Strong	15	32.6
Medium	15	32.6
Weak	16	34.8

### 3.2. Characteristics of the interventions

Characteristics of the included interventions are summarized in Table 2. The total number of study participants ranged from 82 to 18,412 (mean = 1,640). The majority of interventions (81.0%) were multi-component. Thirty one interventions (86.1%) included an educational/informational component, 21 (58.3%) included a cognitive/behavioral component and 28 (77.8%) included a skill development component. Interventions were mainly directed towards secondary school students (91.7%), with the second largest target group being elementary/primary school students (13.9%), followed by college students (2.7%). Intervention sessions varied in length from 5 to 180 min. Duration also varied, ranging from interventions that were administered in an only single session to interventions that were administered over 156 weeks. Length of follow-up also varied from no follow-up to 80 weeks. Further details about each included study and intervention are included in Supplementary Table S1.

**Table 2:** Characteristics of the interventions.

Characteristics of interventions (*n* = 36)		
Number of participants (mean, range)	1,640	82–18,412
School grade^*^	*n*	*%*
Elementary	5	13.9
Secondary	33	91.7
College	1	2.7
Duration (weeks) (mean, range)	22	1 to 156
Length (minutes) (mean, range)	49	5 to 180
Follow-up (weeks) (mean, range)	10	0 to 80
Intervention strategy^*^	*n*	*%*
Educational/Informational	31	86.1
Cognitive/Behavioral	21	58.3
Skills development	28	77.8
Outcomes^*^	*n*	*%*
Cyberbullying	1	2.8
Cyberperpetration	30	83.3
Cybervictimization	32	88.9
Cyberperpetration/Victimization	1	2.8
Cyberbystanding	3	8.3

^*^The total does not necessarily total 36 because the classification system is based on nonexcluding categories.

### 3.3. Risk of bias

**Figure 2:**
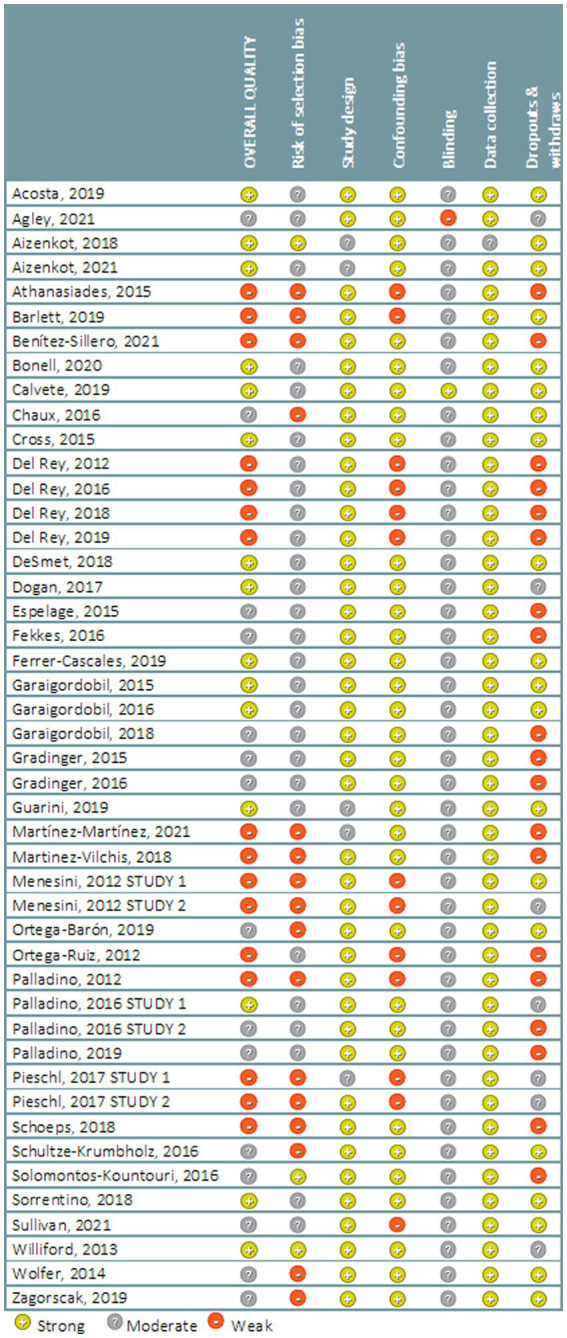
Risk of bias of the included studies.

Data on risk of bias are presented in Figure 2. Fifteen studies were assessed as being of strong methodological quality, whilst 15 were assessed as moderate and also 16 were assessed as weak. Risk of bias was most commonly related with withdrawal and dropout (19 studies), selection bias (18 studies) and confounding bias (13 studies).

### 3.4. Completeness of intervention descriptions

**Figure 3:**
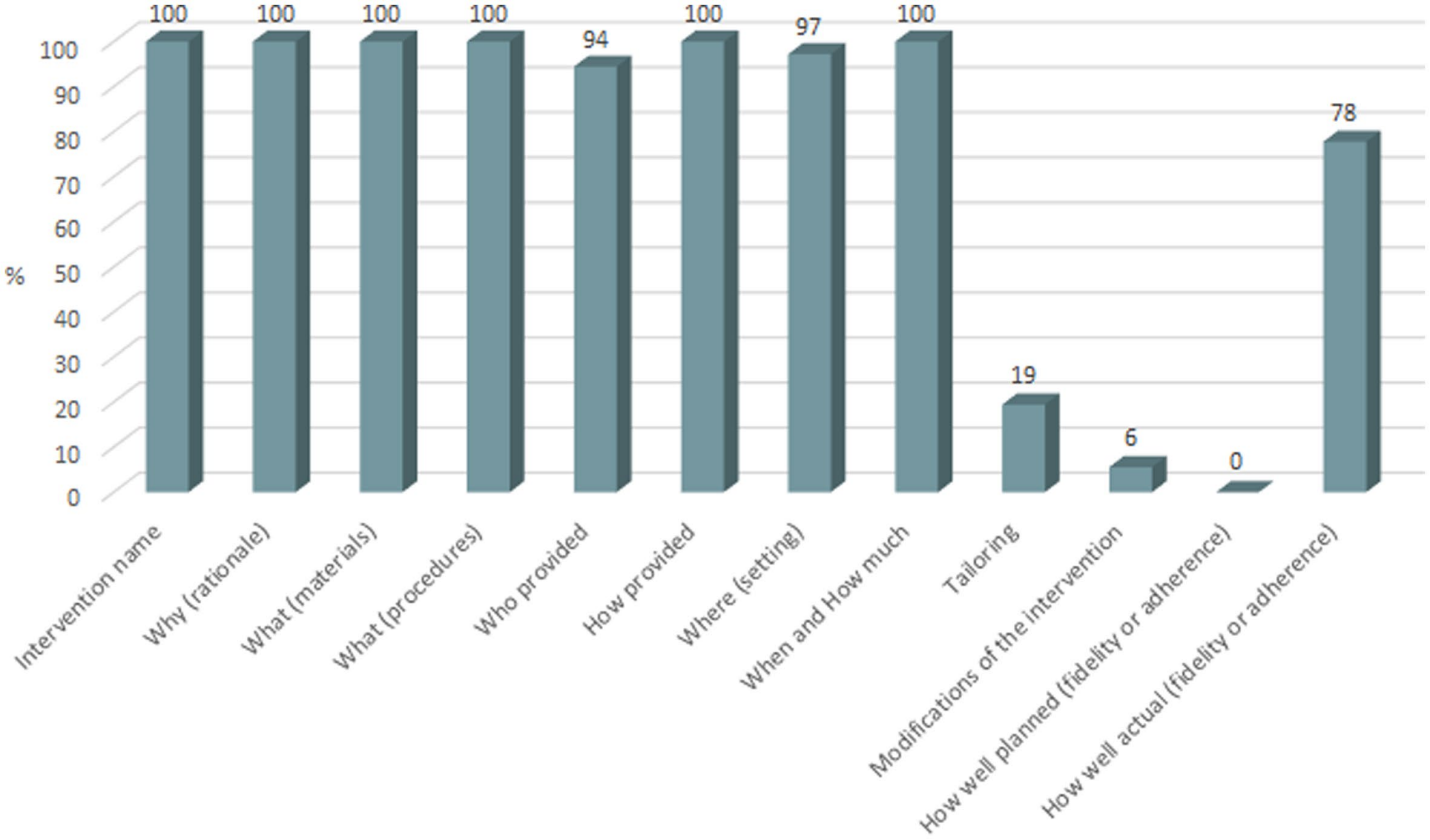
Completeness of the TIDieR checklist.

Included interventions provided different degrees of detail when describing the items considered in the TIDieR checklist. Studies always reported the name and rationale of the included interventions, applied materials and procedures, mode of administration and the duration of sessions delivered by each intervention. 97% of trials reported intervention setting, 94% described who provided the intervention and 78% described the extent to which the intended intervention was actually delivered (fidelity/adherence). In contrast, items such as information related to tailoring (19%), possible intervention modifications (6%) and quality of planning regarding intervention fidelity/adherence (0%) were not described in detail (Figure 3).

### 3.5. Effectiveness

Effectiveness was characterized on the basis of different measured outcomes which could be categorized into the following 5 broad categories: reduction in global CB, reduction in CP, reduction in CV, reduction in CP-CV and reduction in CBS. There were instances in which an intervention was judged to be partially effective. To be classified as partially effective, improvements would be seen, for example, in one particular group but not in others, or at one time-point but not at others.

#### 3.5.1. Reduction of global cyberbullying

The only one intervention measuring reductions in global CB was effective (17). This intervention was multifaceted and included informational/educational, cognitive/behavioral and skill development components but did not include a cognitive/behavioral component.

#### 3.5.2. Reduction of cyberperpetration

Ten of the 33 interventions measuring reduction in CP were effective (18–20, 33–39). Five of these interventions were multifaceted in that they included informational/educational, cognitive/behavioral and skill development components (19, 20, 35, 37, 38). The other five interventions included two components, with Schoeps et al. (39) not including an educational/informational component, Ferrer-Cascales et al. (34), Gradinger et al. (36) and Menesini et al. (18) not including a cognitive/behavioral component, and Del Rey et al. (33) not including a skill development component.

Five interventions were partially effective because they were effective only in particular groups or at certain time-points. The intervention by Bonell et al. (40) was effective at second time-point measurement (36 months) but not at the first one (24 months). The one by Cross et al. (21) was effective at follow-up but not immediately after the intervention. The intervention by Solomontos-Kountouri et al. (41) was effective amongst grade 8 students but not amongst grade 7 students. The one by Sorrentino et al. (42) was effective both overall and amongst males but not amongst females. And the one by Sullivan et al. (43) was effective only after 3 years of continuous implementation. One intervention included all of the three components (43). Three of these interventions were multifaceted including informational/educational and skill development components (21, 41) or cognitive/behavioral and skill development components (40). The other intervention (42) only included an educational/informational component.

Fifiteen interventions were not effective. Twelve of these were multifaceted, of which five included all of the three components (22, 38, 44–46) and seven included two components: The interventions by Del Rey et al. (17), Dogan et al. (47), Menesini et al. (18), and Piesch et al. (48, 49) included educational/informational and skill development components and the ones by Calvete et al. (50) and Del Rey et al. (51) included educational/informational and cognitive behavioral components. The other three interventions included only one component: The one by Barlett et al. (52) included only an educational/informational component, and the interventions by DeSmet et al. (53) and Martínez-Vilchis et al. (24) included only a cognitive/behavioral component.

#### 3.5.3. Reduction of cybervictimization

Eighteen of the 32 interventions measuring CV were effective. Eight of these interventions included all of the three components (19, 35, 37, 38, 44, 45, 54, 55). Other eight included two components: The interventions by Aizenkot et al. (23, 56), Cross et al. (21), Del Rey et al. (17), Ferrer-Cascales et al. (34), Menesini et al. (18), and Solomontos-Kountouri et al. (41) included educational/informational and skill development components, and the one by Del Rey et al. (33) included educational/informational and cognitive behavioral components. The other two interventions included only one component: The one by Athanasiades et al. (57) included only an educational/informational component and the one by Martínez-Vilchis et al. (24) included only a cognitive/behavioral component.

Five interventions were partially effective because they were only effective in particular groups or at certain time-points. The intervention reported by Bonell et al. (40) was effective at second time-point measurement (36 months) but not at the first one (24 months). Interventions described by Gradinger et al. (36) and Sorrentino et al. (42) were effective both overall and amongst males but not amongst females. The intervention reported by Schoeps et al. (39) was effective immediately after the intervention but failed to maintain effects at follow-up. Finally, the intervention described by Sullivan et al. (43) was effective only after 3 years of continuous implementation. This last one included all of the three components while the one described by Sorrentino et al. (42) included only an informational/educational component. The other three interventions included two components: The one by Gradinger et al. (36) included educational/informational and skill development components, and the interventions by Bonell et al. (40) and Schoeps et al. (39) included cognitive/behavioral and skill development components.

Nine interventions were not effective. Two included all of the three components (46, 58). Four included educational/informational and skill development components (18, 47–49) while other two included cognitive/behavioral and skill development components (50, 59). Finally the intervention by DeSmet et al. (53) included only a cognitive/behavioral component.

#### 3.5.4. Reduction of cyberperpetration/victimization

The only one intervention that measured reduction in CP-CV was effective (35) and was multifaceted in that it included informational/educational, cognitive/behavioral and skill development components.

#### 3.5.5. Reduction of cyberbystanding

All of the three interventions measuring reduction of CBS were not effective (35, 49, 53). Two of these interventions were multifaceted with the one by Garaigordobil et al. (35) including all of the three components and the one by Pieschl et al. (49) including informational/educational and skill development components. The intervention by DeSmet et al. (53) only included a cognitive/behavioral component.

## 4. Discussion

This study is, to the best of our knowledge, the first systematic review focused on the effectiveness and nature of the different components of CB interventions that includes students from primary school to college and provides a description of the degree of completeness in which the characteristics of these interventions are reported using the TIDieR check-list.

The present review includes 46 studies reporting on 36 interventions. Fifteen of the included studies obtained a score of “strong” following evaluation of methodological quality, with fifteen obtaining a “moderate” evaluation and sixteen a “weak” evaluation. In relation to the five outcome variables, results were found that varied in nature. The only one intervention measuring reduction in global CB and the one measuring reduction in CP-CV were effective while 50% of those measuring reductions in CP and 72% of those measuring reductions in CV were effective or partially effective. All the intervention measuring reductions in CBS were not effective. When considering effectiveness according to the type of intervention strategy employed, multicomponent interventions that included two or three components represented between 87 and 100%, depending of the measured outcome, of all effective or partially effective interventions.

It is possible that, by not discriminating between CP and CV, the good outcome obtained in the intervention measuring global reduction in CB is due to reduced prevalence of either of these two variables which then modify the global outcome. On the other hand, social desirability is a variable that influences self-declaration as a cyber perpetrator, with low social desirability being found to be a factor associated with greater self-declaration as a cyber perpetrator (60). Therefore, if in the pre-test the number of students self-reporting as CP is lower, given the same effectiveness of the intervention, the observed reduction would be of lesser magnitude. Indeed, equivocal results for CP are found in the intervention that studied both variables and was found to be effective in decreasing CV (17). In the case of interventions that measured differences in the prevalence of CBS, none was found to be effective. This could lead us to suggest that this outcome is due to the difficulty of producing changes in this type of behavior or in the difficulty of objectively measuring these changes (61).

The presence of an “educational/informational” component in more than 80% of interventions that were effective in the reduction of CP highlights the importance of having available appropriate information about CB, its social dynamics and health impact. In the same sense, the presence of a “skill development” component in more than 80% of the interventions that were effective or partially effective in the reduction of CV, also leads us to believe that it is important to offer students the practical tools required to use the Internet in a safe and respectful way, whilst also asking for help when in vulnerable situations. On the other hand, the fact that only near half of the interventions that were effective in the reduction of CV included a “cognitive/behavioral” component, in addition to the good global outcomes obtained by interventions not including this aspect (23, 34, 36) leads us to think that it is probably not a crucial requirement for intervention success. Although it could be more important in interventions aimed at reducing CP since it was present in two thirds of the effective or partially effective interventions for reducing perpetration. Given all of the aforementioned, in addition to the fact that interventions including two or three components turned out to be more effective than those including only one component or approach, it can be concluded that multicomponent interventions are more effective at reducing CB behaviors when they include different strategies (19, 20, 35, 55).

All of the interventions apart from three (52, 56, 58) included secondary school students, making it difficult to compare effectiveness according to educational stages. In addition, only two studies (41, 43) analyzed the influence of school grade in the intervention effectiveness. For this reason, researchers are urged to delve deeper into this aspect in order to be able to understand the importance of adapting interventions according to age or educational level. On the other hand, the only one intervention that included college-aged population (52) was not effective. This leads us to urge the need to deepen research into the effectiveness of this type of intervention within this age group and examine whether interventions shown to be effective amongst secondary school students are applicable here too. Such research is crucial given that CB is also a prevalent issue at these ages and has an influence on the academic, social, and emotional development of these students (62). Only five of the included studies analyzed differences in effectiveness according to sex (19, 36, 40, 42, 43) and although, in general, more positive outcomes were observed amongst males, the lack of studies means that this is another topic in which we must delve deeper. This is especially important given the relevance of gender roles on young people’s behavior (63).

After completion of the TIDieR check-list, included interventions were considered to offer an insufficient level of detail for a number of the analyzed items. This aspect could make the replication of interventions difficult. For example, details in relation to “intervention modifications” were provided within the descriptions of only two of the interventions (44, 59) while details regarding “how well planned” did not appear in the description of any of the interventions and, nevertheless, these are key aspects in intervention development. In not providing information about either of these two dimensions, perhaps because they were not considered within the intervention design or because of word limit restrictions at the time of publishing intervention reports, interpretation of obtained outcomes is more difficult (64). Information pertaining to “tailoring” was also lacking from the majority of write-ups. Huge variety exists in the factors associated with these violent behaviors (65, 66) and, therefore, interventions in different contexts should not be identical. Instead, researchers must consider specific psychosocial and demographic factors (sex, age, school year) at the time of evaluating outcomes or, at least, describe whether or not the intervention was personalized (tailored).

It is important to highlight that a strong point of the present study is that it is the first study to synthesize outcomes according to five groups, whereas other recent studies have not distinguished between cyberaggression and CV (11) or, alternatively, were limited to synthesizing outcomes according to these two categories (4, 26, 27). This approach neglected other categories which are hugely relevant to understanding the complex phenomenon of CB. Further, it is the first SLR to include university students within its study sample and is also the first published work to have analyzed the completeness of descriptions provided regarding intervention characteristics. This has enabled typically insufficiently described items to be detected which is important as this makes later replication of interventions more difficult.

As main limitations of the present study, it is important to indicate that according to our exclusion criteria, studies that did not offer as an outcome changes in the in the prevalence of any of the dimensions of CB were excluded, the articles that evaluated the effectiveness of the interventions to change the attitude of bystanders in relation to CB events were not taken into account for this review (67). Moreover, the lack of studies included in comparison to other recent SLRs, which evaluated intervention programs targeting traditional bullying (27, 68). This is a topic that has been studied over many years and has, therefore, generated a much greater quantity of literature.

## 5. Conclusion

CB is a highly diffuse study area with non-tangible limits given the large number of routes through which it can take place and the fact that it is a private behavior in nature. As a result, more dramatic cases to have had strong implications and have been picked up by the media have been in relation to traditional bullying, with this having awaken greater interest in researchers. Nonetheless, as seen in everything previously discussed, the health consequences of CB can be enormous and the number of affected adolescents (minors) beyond measure. It grabs the attention, therefore, that more effort is not being invested in this aspect when it is a well-recognized public health issue. Tackling the issues described here do not appear to be a priority of political agendas.

Given the aforementioned, it is critical to increase the number of studies and the quality of interventions targeting CB in order to obtain more robust outcomes about how to reduce its prevalence. Future research should be directed towards:

1. Deepen knowledge on the effectiveness of interventions within the university population and conduct differentiated analysis according to age groups and sex.
2. Increase the precision and robustness of obtained outcomes. This could be achieved through the use of RCT designs and broader samples, as suggested in the SLR conducted by Gaffney (27).
3. Reduce the most common types of bias seen in different studies. CONSORT guidelines may be of use for carrying out high quality RCTs (69).
4. Provide detailed and complete information about the way in which interventions are carried out. In this sense, the TIDieR checklist may provide a great support to researchers, helping them include all of the details that are relevant to their interventions (32).

## Data availability statement

The original contributions presented in the study are included in the article/Supplementary material, further inquiries can be directed to the corresponding author.

## Author contributions

JH-M and IR-P: conceptualization. JH-M, AR-S, IR-P, MR-G, and GP-M: methodology and writing—original draft preparation. JH-M: software. AR-S, IR-P, MR-G, and GP-M: validation. JH-M and IR-P: resources. All authors contributed to the article and approved the submitted version.

## Funding

This work and the article processing charge was funded by Carlos III Health Institute (ISCIII) (PI20/01018) and the Minstry of Health of Andalusia (PE-0221-2018) and co-funded with FEDER and FSE+ funds.

## Conflict of interest

The authors declare that the research was conducted in the absence of any commercial or financial relationships that could be construed as a potential conflict of interest.

## Publisher’s note

All claims expressed in this article are solely those of the authors and do not necessarily represent those of their affiliated organizations, or those of the publisher, the editors and the reviewers. Any product that may be evaluated in this article, or claim that may be made by its manufacturer, is not guaranteed or endorsed by the publisher.
