# Supplementary material for: Characteristics and effectiveness of interventions to reduce cyberbullying: a systematic review
Source: Front Public Health. 2023 Aug 30;11:1219727. doi: 10.3389/fpubh.2023.1219727 (PMC10498774; doi:10.3389/fpubh.2023.1219727)
Supplement: Supplementary file 1 [file Data_Sheet_1.docx]

Supplementary Material

Characteristics and Effectiveness of Interventions to Reduce Cyberbullying: A Systematic Review

# Supplementary Data

**Full search strategy and Register**

| **Databases** | **Platform/ Access** | **Search date** | **References retrieved** |
| --- | --- | --- | --- |
|  | | | |
| Medline | Pubmed | 14/2/2022 | 1629 |
| Scopus |  | 14/2/2022 | 1885 |
| ERIC |  | 14/2/2022 | 468 |
| Psycinfo |  | 14/2/2022 | 1901 |
|  | | | |
| TOTAL with duplicates |  |  | 5853 |
| Duplicates |  |  | 2376 |
| TOTAL |  |  | 3477 |

**Search Strategy:**

(Cyberbullying OR cyber-bullying OR cyberaggression OR “cyber aggression” OR cybervictimization OR “cyber victimization” OR “cyber victim” OR “cyber harassment” OR "cyber bullied" OR sexting OR morphing OR oversharing OR flaming OR phishing OR phubbing) AND (Adolescen* OR teenager* OR teen* OR "young person" OR "young persons" OR "young adults" OR "young adult" OR "young people" OR Youth OR child* OR undergraduates  OR  "college student*"  OR  "university graduate*"  OR  "university student*")

**Psyinfo**

su(Cyberbullying OR cyber-bullying OR cyberaggression OR "cyber aggression" OR cybervictimization OR "cyber victimization" OR "cyber victim" OR "cyber harassment" OR "cyber bullied" OR sexting OR morphing OR oversharing OR flaming OR phishing OR phubbing) AND su(Adolescen* OR teenager* OR teen* OR "young person" OR "young persons" OR "young adults" OR "young adult" OR "young people" OR Youth OR child* OR undergraduates OR "college student*" OR "university graduate*" OR "university student*")

**ERIC**

noft(Adolescen* OR teenager* OR teen* OR "young person" OR "young persons" OR "young adults" OR "young adult" OR "young people" OR Youth OR child* OR undergraduates OR "college student*" OR "university graduate*" OR "university student*") AND noft(Cyberbullying OR cyber-bullying OR cyberaggression OR "cyber aggression" OR cybervictimization OR "cyber victimization" OR "cyber victim" OR "cyber harassment" OR "cyber bullied" OR sexting OR morphing OR oversharing OR flaming OR phishing OR phubbing)

**Pubmed**

(("Cyberbullying"[Text Word] OR "cyber-bullying"[Text Word] OR "cyberaggression"[Text Word] OR "cyber aggression"[Text Word] OR "cybervictimization"[Text Word] OR "cyber victimization"[Text Word] OR "cyber victim"[Text Word] OR "cyber harassment"[Text Word] OR "cyber bullied"[Text Word] OR "sexting"[Text Word] OR "morphing"[Text Word] OR "oversharing"[Text Word] OR "flaming"[Text Word] OR "phishing"[Text Word] OR "phubbing"[Text Word]) AND ("adolescen*"[Text Word] OR "teenager*"[Text Word] OR "teen*"[Text Word] OR "young person"[Text Word] OR "young persons"[Text Word] OR "young adults"[Text Word] OR "young adult"[Text Word] OR "young people"[Text Word] OR "Youth"[Text Word] OR "child*"[Text Word] OR "undergraduates"[Text Word] OR "college student*"[Text Word] OR "university graduate*"[Text Word] OR "university student*"[Text Word]))

**Scopus**

( KEY ( adolescen* OR teenager* OR teen* OR "young person" OR "young persons" OR "young adults" OR "young adult" OR "young people" OR youth OR child* OR undergraduates OR "college student*" OR "university graduate*" OR "university student*" ) AND KEY ( cyberbullying OR cyber-bullying OR cyberaggression OR "cyber aggression" OR cybervictimization OR "cyber victimization" OR "cyber victim" OR "cyber harassment" OR "cyber bullied" OR sexting OR morphing OR oversharing OR flaming OR phishing OR phubbing ) )

# Supplementary Table

**Supplementary Table 1**. Characteristics of the included interventions

| **Author/ Year / Country** | **Aim of the intervention** | **Study design /N/ Follow-up** | **Target population**  **(grade and age)** | **Intervention vs. Control** | **Components of the intervention arms** | **Measurements instruments** | **Main outcomes** |
| --- | --- | --- | --- | --- | --- | --- | --- |
| **Acosta et al.**  **2019**  **USA.** | 1.- Reduce Cybervictimization | Randomized controlled trial/2771 /No | Students at 13 middle schools evenly split between grades 6 (48%) and 7 (52%), and primarily of ages 11 (38%) or 12 (41%). The schools were located throughout Maine, USA. | A.- Intervention group (977 students): 7 schools (initially 8 but one school dropped out of the study because of data privacy concerns) received the Restorative Practices Intervention.  B.- Control group (1794 students): six schools, no intervention.  Intervention and control schools were matched based on demographic, academic, and disciplinary data, and then randomized. | A.- Intervention program:  **- Duration:** 2 years.  **- Format:** the survey was completed via computer in classrooms under the supervision of teachers.  Training, monthly consultation, and ongoing participatory learning groups were used to support and monitor the implementation, and International Institute of Restorative Practices coaches visited campuses twice per year to troubleshoot on-site.  **- Content:**  Essential restorative practices:  1. Affective statements  2. Restorative questions  3. Small impromptu conferences  4. Proactive circles (comprise at least 80% of circles conducted at school)  5. Responsive circles (comprise no more than 20% of circles conducted at school)  6. Restorative conferences  7. Fair process  8. Reintegrative management of shame  9. Restorative staff community  10. Restorative approach with families  11. Fundamental hypothesis  B.- No intervention | 1.- Cybervictimization:  Three items from the Communities That Care Survey (Arthur et al. 2007). | Results were not significant for any of the 11 analyzed outcomes, including cyberbullying victimization, predicted by intervention condition:  1.- CV Pre-Post: (OR (95%CI):  OR = 0.89, 95% CI = 0.50-1.59, p > 0.05 |
| **Agley et al.**  **2021**  **USA** | To reduce cyberbullying both:  1.- victimization behavior  2.- perpetration behavior | Randomized controlled trial/1594/No | Students at grades 4, 7, and 10. | A. Intervention: ACT Out! Social Issue Theater (774).  B. Control: Treatment as usual (763). | ACT Out! Social Issue Theater:  **-Duration**: 1 hour  **-Format**: psychodramatic, improvisational performance  **-Content**: Each 1-hour performance consisted of 5 vignettes focused on bullying and cyberbullying and was designed to be interactive; after each scenario, the student audience was invited to converse with the performers, who remained in character  B.- No intervention | 1/2.- Bullying and Cyberbullying Scale for Adolescents  (BCS-A) | 1.- CV: Mean (SE):  Control: Pre-Post: 0.47 (0.5) – 0.44 (0.5); p=0.292  Intervention: Pre-Post: 0.63 (0.5) – 0.50 (0.5); p<0.001  2.- CP: Mean (SE):  Control: Pre-Post: 0.25 (0.4) – 0.27 (0.4); p=0.388  Intervention: Pre-Post: 0.27 (0.4) – 0.31 (0.4); p=0.210 |
| **Aizenkot et al.**  **2018**  **Israel** | 1.- Reduce Cybervictimization in WhatsApp classmates’ groups. | QE /1402/ No | Students from 4^th^ to 6^th^ grades and 8^th^ grade (10-14 years old). | A.- Experimental group: Intervention program. | A.- Intervention program:  -**Duration**: 8 weekly lessons (1 hour per lessons).  -**Format:** Short videos, interactive activities, group discussion cards…  -**Content:** Knowledge and competencies, Attitudes toward WhatsApp online cyberbullying, Subjective and social norms and Perceived behavioral control.  All the lessons included an informative part at the beginning and then group activities and discussions later on. | 1.- Cyberbullying in WhatsApp classmates’ group questionnaire.  . | Differences between pre-post intervention scores (Wilcoxon/Mann-Withney):  1.- Cybervictimization total scores:  -Degree of cybervictimization: t1=10.18 (5.41), t2=9.23 (5.17); Z=-2.29, p<0.05.  -Cybervictimization (% of students): t1=64.22 (16.61), t2=59.64 (14.05); Z=-2.68, p<0.05  Results after controlling for grade:  -Cybervictimization total score: F(1, 50) = 4.56, p <0.05  -Cybervictimization (class %): F(1, 50) = 2.93, p <0.05 |
| **Aizenkot et al.**  **2021**  **Israel** | 1.- Reduce Cybervictimization in WhatsApp classmate discourse | Non-RCT/533/No | Students from 4^th^ to 6^th^ grades (10-12 years old). | A. Intervention. Safe Surfing intervention program (391).  B. Control group: As usual (142). | A.- Safe Surfing intervention program:  **-Duration:** eight weekly lesson (1h)  **-Format**: short videos, dilemma-based stories, interactive activities, group discussion, cards and information provided and discussed (news items and relevant laws). All lessons began with an informative segment followed by  group activities and a discussion.  **-Content**: topics: understanding the definition of cyberbullying; expressions and implications of cyberbullying in general, and WhatsApp cyberbullying in  particular; the role of bystanders, including developing personal and mutual responsibility; acquaintance with state laws prohibiting cyberbullying; developing skills for judging and self-monitoring contents before disseminating them online; and formulating school rules in workshops attended by the entire school population (teachers, students, and parents). Each topic was covered in either one or two lesson plans.  B.- No intervention | 1.- Questionnaire “cyberbullying victimization in WhatsApp classmate discourse” | 1.- CV (Two-way ANOVAs):  The experiment group demonstrated  significantly decreased cyberbullying victimization in WhatsApp classmate group discourse, z(385) = 4.59, p = .001, r = .40, while the control group showed a significant increase, z(141) = 3.35, p = .001, r = .82. |
| **Athanasiades et al.**  **2015**  **Greece** | 1.- Reduce Cybervictimization. | RCT/314/6 months | Students from second grade of middle school (13-14 years old). | A.- Experimental group: The TABBY project intervention (123).  B.- Control group: No intervention (140). | A.- Intervention program:  -**Duration**: 2 hours.  -**Format**: Four videos (each one negotiating a different form of cyberbullying), followed by a discussion.  -**Content**: Discussion on the negative consequences of cyberbullying as well as on proper internet use and certain actions against cybervictimization.  B.- No intervention. | 1.- The TABBY checklist. | 1.- Cybervictimization in the next 6 months (Mean (SD); ANOVA):  -Experimental group: t1=1.27 (1.50), t2=0.88 (1.39); p<0.01  -Control group: nonsignificant.  1.- Cybervictimization in the last 6 months (Mean (SD); ANOVA):  -Experimental group: t1=0.76 (1.03), t2=0.51 (0.88); p<0.05  -Control group: nonsignificant. |
| **Barlett et al.**  **2019**  **USA** | 1.- Reduce Cyberperpetration. | CCT/126/2 months | Students from an arts college in their first or second year of undergraduate training (18-20 years old). | A.- Experimental group: Intervention video (n???).  B1.- Control group: Control video (n???).  B2.- Control group: No video. | A.- Intervention video:  -**Duration**: 10-15 min per video.  -**Format**: Short online videos.  -**Content**: 3 videos indicating that individuals are not as anonymous online as they may believe.  B1.- Control video:  -**Duration**: 10-15 min per video.  -**Format**: Short online videos.  -**Content**: 3 videos whose theme was how online communication often leads to  Misunderstandings.  B2.- No video. | 1.- The Malice subscale of the Cyberbullying Experiences Survey. | 1.- Cyberbullying perpetration (Mixed ANOVA):  A nonsignificant main effect of wave, a nonsignificant main effect of group and a nonsignificant two-way interaction. |
| **Benítez-Sillero et al.**  **2021**  **Spain.** | To prevent cyberbullying both:  1.- Cybervictimization  2.- Cyberperpetration | Quasi-experimental / 764 / No. | Students 12–19 years, first to fourth years of secondary education  (12–16 years old) and first and second years of high school (17–18 years old). | A. Experimental group: PRE-BULLPE (439).  B. Control group (325). | A.- PRE-BULLPE:  **-Duration:** six sessions of physical education class lasting 1 h each.  **-Format**: The contents were adapted to the methodological strategies of physical education and its curriculum, including the following: cooperative games or challenges; body expression, mainly dramatization; ‘locomotor story’ that is tale where a story is told and the students represent the actions with movement; awareness and body limitation activities; motor games with symbolic roles; relay games emphasizing respect for rules; and competitive games adapted by changing roles  **-Content**: The psychosocial contents developed were as follows: knowledge of bullying, the roles of victim and aggressor, knowledge and expression of basic emotions, importance of the social group, collaborative work, self-esteem, empathy, self-control, resilience and discrimination.  B.- No intervention. | 1/2.- Spanish version of the  European Cyberbullying Intervention Project Questionnaire (ECIPQ) | The differences between the control and quasi-experimental groups and between pre-test and post-test were analyzed using a repeat measurement analysis for related samples.  1.- CV (differences in the pre-test and post-test measures):  In the quasi-experimental group, the cyberbullying victimization variable decreased (F = 6,34; p = .013) to a greater extent than it did in the control group after the intervention.  2.- CP (differences in the pre-test and post-test measures):  The same difference was not found in the cyberbullying aggression variable (F = 0,099; p = .753). |
| **Bonell et al.**  **2020**  **UK.** | 1.- Reduce Cybervictimization.  2.- Reduce Cyberperpetration. | Cluster randomized controlled trial / 6667 students / No. | Students aged 11/12 years (end of year 7) at baseline. | A.- Experimental group (20 schools): ‘Learning Together’ intervention  B.- Control group (20 schools): No intervention. | A.- ‘Learning Together’ Intervention program:  **- Duration:** 3 years.  **- Format:** Schools delivered a social and emotional skills curriculum for students in years 8–10, in total 5–10h of teaching per year.  School action groups comprising at least six staff and six students, led by a member of the school’s senior leadership team, were created to review anonymized findings from the school’s baseline survey to understand local needs. The groups aimed to coordinate the intervention and revise policies so that these supported the use of restorative practices.  **- Content:** teaching of a social and emotional skills curriculum for students in years 8–10. The curriculum addressed bullying and aggression but not specific to a particular setting such as school or online.  B.- No intervention. | 1/2.- Cyberbullying victimization: adapted from Smith and colleagues’ measure (Smith, 2008). | At baseline:  1.- Cyberbullying victimization:  Control, n (%) = 522 (16.0)  Intervention, n (%) = 467 (14.5)  2.- Cyberbullying perpetration:  Control, n (%) = 290 (8.9)  Intervention, n (%)= 279 (8.6)  At 24 months:  1.- Cyberbullying victimization:  Control: n/N (%) = 443/3116 (14.2%)  Intervention: n/N (%) = 340/2993 (11.4%)  Unadjusted OR (95% CI) = 0.79 (0.63-0.99), p = 0.041.  Adjusted OR (95% CI) = 0.77 (0.61-0.98), p = 0.035.  2.- Cyberbullying perpetration:  Control: n/N (%) = 257/3116 (8.3%)  Intervention: n/N (%) = 229/2984 (7.7%)  Unadjusted OR (95% CI) = 0.93 (0.71-1.21), p = 0.572.  Adjusted OR (95% CI) = 0.90 (0.67-1.19), p = 0.450.  At 36 months:  1.- Cyberbullying victimization:  Control: n/N (%) = 347/2987 (11.6%)  Intervention: n/N (%) = 266/2754 (9.7%)  Unadjusted OR (95% CI) = 0.83 (0.65-1.05), p = 0.121.  Adjusted OR (95% CI) = 0.80 (0.62-1.05), p = 0.110.  2.- Cyberbullying perpetration:  Control: n/N (%) = 287/3008 (9.5%)  Intervention: n/N (%) = 193/2766 (7.0%)  Unadjusted OR (95% CI) = 0.66 (0.51-0.87), p = 0.003.  Adjusted OR (95% CI) = 0.65 (0.48-0.88), p = 0.005.  In summary, at 24 months comparing intervention with control schools, they found lower rates of cyberbullying victimization but not perpetration. At 36 months, they found reduced rates of: cyberbullying perpetration but not victimization. Besides, intervention effects on cyberbullying perpetration at 24 months were moderated by student sex, such that effects were larger for boys. |
| **Calvete et al.**  **2019**  **Spain.** | To reduce the reciprocity between:  1. Cybervictimization  2.- Cyberperpetration | RCT/858 students/12 months | High school students aged 12 to 17. | A.- Experimental group: Incremental Theory of Personality Intervention (ITPI) (452).  B.- Control group: containing scientific information and information on the human brain (406) | A.- ITPI:  **- Duration:** divided in three parts with a total duration of 50–60 min.  **- Format:** students completed the intervention and assessment in their classrooms during school hours. The session was administered by a trained psychology research assistant.  **- Content:**  1. Participants read a scientific article with information about individuals´ potential to change.  2. Participants read extracts written by other students that had participated in the study and written their own conclusions.  3. Participants described a time when they felt isolated, rejected, or disappointed by another person at school. Next, they imagined that the same event happened to another student and wrote 1-3 paragraphs describing what they could do or say to help the other student to understand that people can change and the things happening to him/her might also change.    B. Control group  **- Duration:** divided in three parts with total duration of 50–60 min.  **- Format:** students completed the session and assessment in their classrooms during school hours. The session, administered by a trained psychology research assistant.  **- Content:**  1. Participants read a scientific article about the brain, including brain localization and the role of different brain areas in supporting cognitive functioning. 2. Participants read stories written by other adolescents explaining how they became accustomed to the sensory and physical environment of high school.  3. Participants wrote about how and why students adapt to the physical environment at high school. | 1/2.- Cyberbullying Questionnaire (CBQ) | Differences in the comparison with control group by t tests:  1.- Cyberbullying victimization:  T1: control M (SD) T1 = 1.34 (2.40) vs. intervention M (SD) T1 = 1.42 (2.45), t = 0.53, p = 0.596.  T2: control M (SD) T2 = 1.39 (2.88) vs. intervention M (SD) T2 = 1.22 (2.81), t = -0.83, p = 0.406.  T3: control M (SD) T3 = 1.24 (2.84) vs. intervention M (SD) T3 = 1.26 (3.25), t = 0.09, p = 0.929.  2.- Cyberbullying perpetration:  T1: control M (SD) T1 = 1.01 (1.98) vs. intervention M (SD) T1 = 1.21 (2.35), t = 1.35, p = 0.178.  T2: control M (SD) T2 = 1.31 (3.22) vs. intervention M (SD) T2 = 1.13 (2.86)., t = -0.82, p = 0.413.  T3: control M (SD) T3 = 1.13 (3.20) vs. intervention M (SD) T3 = 1.09 (3.11), t = -0.17, p = 0.862. |
| **Cross et al.**  **2016**  **Australia** | 1.- Reduce Cybervictimization  2.- Reduce Cyberperpetration | RCT/3382/28 months | Students enrolled in 8^th^ and 9^th^ grade (13-14 years old). | A.- Experimental Group: Cyber Friendly School Program (CFSP) (1582)  B.- Control Group: No intervention (1292) | A.- Cyber Friendly School Program (CFSP)  -**Duration**: 24 months.  -**Format**: Online resources. Interactive activities such as problem solving, quizzes, and case studies were also provided to consolidate student learning in each module.  -**Content**: Whole-school level program: strategies to develop: students’ social relationships and peer support; policy and its implementation involving the school community; school ethos; student social and emotional development; positive behavior management with fewer punitive solutions; and more school–home-community links.  Student cohort program: Reduce student harm via “5Cs:” (i) the online contexts where students spent time; (ii) the online contacts they made; (iii) how they managed their confidentiality (privacy); (iv) their conduct and online skills; and (v) the content they accessed.  B.- No intervention. | 1/2.- Two 11-item scales, based on that of Smith, Mahdavi, Carvalho, and Tippett (2006) and formative work with young people conducted by the research team. | 1.- Cybervictimization:  Control: t1=73% (0.08 (0.25)); t2=74% (0.11 (0.33)); t3=76% (0.13 (0.46)).  Experimental: t1=70% (0.10 (0.26)); t2=75% (0.10 (0.31)); t3=77% (0.10 (0.33)).  **Significant increase** of the non-involved students’ percentage among the experimental group. T = 2.124 p= .034  1.- Cyberperpetration:  Control: t1=91% (0.02 (0.12)); t2=90% (0.03 (0.14)); t3=93% (0.03 (0.25)).  Experimental: t1=89% (0.03 (0.17)); t2=92% (0.03 (0.26)); t3=93% (0.03 (0.22)).  **Not significant increase** of the non-involved students’ percentage among the experimental group. T = .962 p = .336  Only significant during follow-up. T = 2.725 p = .006 |
| **Del Rey et al.**  **2012**  **Spain** | 1.- Reduce Cyberbullying  2.- Reduce Cyberperpetration  3.- Reduce Cybervictimization | CCT/893/No | Students from secondary school (11-19 years old). | A.- Experimental group: The ConRed Program (595).  B.- Control group: No intervention (298). | A.- The ConRed Program.  -**Duration**: 3 months.  -**Format**: training sessions were  held with teachers and the families of schoolchildren, with the schoolchildren themselves forming the principal target group.  -**Content**: a) Internet and social networks; b) benefits of Internet use and instrumental skills and c) risks and advice on usage.  B.- No intervention. | 1/2/3.- European Cyberbullying  Intervention Project Questionnaire (ECIPQ). | Changes in Cyberbullying (Student’s T-test):  1.- Cyberbullying  Control: nonsignificant.  Experimental: t=2.717; p<0.05  2.- Cyberperpetration:  Control: nonsignificant.  Experimental: nonsignificant  3.- Cybervictimization:  Control: nonsignificant.  Experimental: t=2.726; p<0.05 |
| **Del Rey et al.**  **2018**  **Spain** | 1.- Reduce Cyberperpetration | CCT/479/No | Students in secondary school (12-18 years old). | A.- Experimental group: Implementation of the ‘Asegúrate” program (292).  B. Control group: No intervention (implemented after post-test) (187). | A.- ‘Asegúrate’ program:  -**Duration**: At least four modules during a three months period.  -**Format**: presentation sessions.  -**Content**: (a) The theory of normative social behaviour.  b) Self-regulation skills.  c) The ideas/beliefs held by adolescents.  B.- No intervention. | 1.- European Cyberbullying Intervention Project Questionnaire (ECIPQ). | 1.- Reduce Cyberperpetration (chi-cuadrado):  -Control group: +52%; x^2^ (1, 187)=24.028 p=0.001.  -Experimental group: -17.5%; nonsignificant. |
| **Del Rey et al. 2019**  **Spain** | Reduce Cyberbullying:  1.- Cybervictimization  2.- Cyberperpetration | Non-randomized Trial/4779/No | Students in 5^th^ 6^th^ grades of primary education and secondary education (10-16 years old). | A.- Experimental Group: ‘Asegúrate program’ (2316)  B.- Control Group: No intervention (2436) | A.- ‘Asegúrate’ program:  -**Duration**: 8 sessions.  -**Format**: presentation sessions with audiovisual material and internet resources.  -**Content**: (a) “Trending topic” explores the current ideas held by the participants; (b) “My profile” encourages the participants to reflect on any of their own activities on the social networks which might be described as “not normal”; (c) “Stop to think” focuses on analyzing the reasons which lead us, or others, to behave in certain ways when using social networks; (d) “Like/Don’t like” identifies the possible consequences of their own (and others’) positive or negative behavior on social networks; and (e) “I share” allows each session to end with a conclusion and an individual and/or collective declaration of commitment.  B.- No intervention. | 1/2.- European Cyberbullying  Intervention Project Questionnaire (ECIPQ). | 1.- Changes in Cybervictimization (Mean (SD)):  -Control: t1=0.10 (0.19); t2=0.10 (0.22)  -Experimental: t1=0.15 (0.25); t2=0.12 (0.25)  p<0.05  2.- Changes in Cyberperpetration (Mean SD)):  -Control: t1=0.06 (0.15); t2=0.06 (0.16)  -Experimental: t1=0.09 (0.18); t2=0.06 (0.19)  p=0.001 |
| **DeSmet et al.**  **2018**  **Belgium** | 1.- To reduce negative bystander behavior on Cyberbullying perpetration  and victimization | Cluster RCT /238/4-week follow-up | Students in 8^th^ grade classes (13-14 years old). | A. Experimental group: serious digital game (129).  B: Control group: No intervention (109).  one week prior to the intervention (T0), immediately after the intervention or one week after T0 for the control group (T1), and at 4-week follow-up (T2). | A.- Intervention program: intervention to increase positive bystander behavior and decrease negative bystander behavior in cyberbullying among young adolescents.  -**Duration**: Once  -**Format**: A game.  -**Content**: The player in the story is transferred from the future to this school in the year 2015, to solve this problem by talking to pupils, responding to cyberbullying by using positive bystander behavior, and thus help end the cyberbullying and get the ‘ugly people page’ removed.  B.- No intervention. | 1.- Bullying and cyberbullying prevalence (perpetration, victimization, bystanding) questionnaire.  Cyberaggresion, Cybervictimization and Bystanders. | RM ANOVA:  1.- The intervention also showed a potentially undesired effect: effect of witnessing cyberbullying decreased in both conditions, but decreasing was stronger in the control condition F(2, 377)= 4.72; (p<0.05). Cybervictimization and Cyberaggression: nonsignificant. |
| **Dogan et al.**  **2017**  **Turkey** | Reduce Cyberbullying:  1.- Cyberperpetration.  2.- Cybervictimization | QE/642/5 months | Students in 5th grade (10-12 years old). | A1.- Experimental group: The ViSC Social Competence Program (School and class level) (227).  A2.- Experimental group: The ViSC Social Competence Program (School level) (201).  B.- Control group: No intervention (214). | A1.- School and class level intervention:  -**Duration**: 90 minutes per unit (class level).  -**Format**: a 13-unit class project is implemented by teachers in their classes class level).  -**Content**: work on how to prevent bullying in their classroom and work together on creating a common activity (class level)  A2.- School level intervention.  -**Duration**: 3-hour teacher-training seminar.  -**Format**: a series of in-school trainings for teachers are organized by so called ViSC coaches.  -**Content**: to recognize and differentiate bullies, victims, and bully-victims; and to conduct structured conversations with them.  B.- No intervention. | 1/2.- Cyberbullying and Cybervictimization scales containing one global and  seven specific items related to different electronic  means based on Smith and colleagues (2008). | 1.- Cyberperpetration: Mean (SD):  -School and class level intervention: t1=0.03 (0.19), t2=0.04 (0.11), t3=0.06 (0.16).  -School level intervention: t1=0.05 (0.20), t2=0.07 (0.24), t3=0.05 (0.25).  -Control group: t1= 0.06 (0.22), t2=0.08 (0.25), t3=0.06 (0.21).  2.- Cybervictimization: Mean (SD):  -School and class level intervention: t1=0.10 (0.33), t2=0.15 (0.29), t3=0.11 (0.27).  -School level intervention: t1=0.10 (0.26), t2=0.18 (0.40), t3=0.11 (0.27).  -Control group: t1= 0.15 (0.33), t2=0.14 (0.36), t3=0.11 (0.29). |
| **Espelage et al.**  **2015**  **USA** | 1.- Reduce Cyberperpetration. | RCT/3651/No | Students in 6^th^-8^th^ grade | A.- Experimental group: Second Step Middle School Program (1941).  B.- Control group: No intervention (1710). | A.- Second Step Program.  -**Duration**: 15 lessons at Grade 6 and 13 lessons at Grades 7 and 8. Lessons are delivered in one 50-min or two 25-min classroom sessions, taught weekly or semiweekly throughout the school year.  -**Format**: structured lessons supported  through an accompanying DVD, which contains media-rich content.  -**Content**: lessons on empathy, communication, bullying, emotion regulation, problem solving, substance abuse prevention.  B.- No intervention | 1.- A four-item scale based on the work of Ybarra, Espelage, and Mitchell (2007). | 1.- Changes in Cyberperpetration:  Second Step 🡪 Cyberperpetration= nonsignificant. |
| **Fekkes et al.**  **2016**  **The Netherlands** | 1.- Reduce Cybervictimization | Cluster-CCT/1394/20 months | Students from grades 7-9 (13-16 years old). | A.- Experimental group: Skills for life program (913).  B.- Control group: No intervention (481). | A.- Skills for life program:  -**Duration**: 25 lessons (during 2 school years).  -**Format**: different teaching methods including active enactment, modelling with the use of DVD extracts, role play, discussion, feedback and making commitments to engage in healthy behavior.  -**Content**: Awareness and handling of thoughts, feelings, interpersonal problem solving skills, emotion regulation skills, critical thinking, giving and seeking help, dealing with bullying, setting and respecting boundaries, substance use, norms, values and friendships, sexuality, suicidal thoughts and conflicts with teachers and peers.  B.- No intervention | 1.- Self-report questionnaires derived from questionnaires used in previous studies. | 1.-Changes in cybervictimization:  Experimental: t1=2%, t2=4%, t3=5%.  Control: t1=4%, t2=1%, t3=7%.  Effects at t2: OR=20.19; 95%CI (1.20-338.92).  Effects at t3: OR=4.73; 95%CI (0.62-35.97).  Adjusted for age, gender, educational level and urbanization. |
| **Ferrer-Cascales et al.**  **2019**  **Spain.** | Reduce Cyberbullying both:  1.- Cyberperpetration  2.- Cybervictimization | RCT/2057 students randomly assigned to the experimental group or the control group/No | Students of secondary education (aged 11 to 16 years). | A.- Experimental group: received the “Tutoría Entre Iguales” (TEI) (987).  B.- Control group: No intervention (1070). | A.- TEI program:  **- Duration:** 1 hour per session a whole academic year (9 months)  **- Format:** six stages. In particular, the program was based on tutorials between tutor-tutee students. Besides, other group activities were conducted.  **- Content:**  Stage 1: Dissemination and Awareness about the Intervention along the School Community.  Stage 2: Teacher training, 30 h (10 h face-to-face format and 20 h virtual format).  Stage 3: Student Tutors Training, initial training of 3 sessions lasting 1 h.  Stage 4: Pairing Students. An interview between tutors and tutees was conducted, and group dynamics during this session were developed to promote cooperativeness between them.  Stage 5: Intervention Development, included cohesion activities, monthly tutorial activities, specific training activities.  Stage 6: Closing, a joint activity performed at the end of the academic year. | 1.- E-Bullying Scale (E-BS  2.- E-Victimization Scale (E-VS) | The percentage change between scores from T1 to T2 was calculated following this formula: [(T2T1/T1)]*100. Age was introduced in the analyses as a covariate.  1.- Cyberperpetration (ANCOVA):  Control group: M (SD) T1 = 2.08 (3.13), M (SD) T2 2.21 (4.48), % change 6.25%.  Intervention group: M (SD) T1 = 2.27 (3.10), M (SD) T2 1.59 (3.80), % change -29.95%.  2.- Cybervictimization (ANCOVA):  Control group: M (SD) T1 = 2.95 (5.10), M (SD) T2 2.70 (5.30), % change -8.47%.  Intervention group: M (SD) T1 = 3.19 (4.82), M (SD) T2 1.94 (4.51), % change -39.18%.  The involvement in cyberbullying / cybervictimization decreased over time only in the experimental group. |
| **Garaigordobil et al.**  **2015**  **Spain** | 1.- Reduce cyberbullying:  1.- Cybervictimization  2.- Cyberperpetration  3.- Cyberbystanding  4.- Cyberaggression-Victimization | Randomized Controlled Trial/176/0 | Students enrolled in 7^th^ to 10^th^ grades (13-15 years old) | A.- Experimental Group: Cyberprogram 2.0 (93)  B.- Control Group: No intervention (83) | A.- Cyberprogram 2.0:  -**Duration**: 19 one-hour sessions carried out during the school term.  -**Format**: The sessions begin with the group members sitting in a circle on the floor. The adult explains the activity, its goals, and so forth, and the participants carry out the action. Subsequently, the adult leads a discussion and guided reflection, promoting critical reflection by asking nonjudgmental questions. The program uses diverse group dynamic techniques to stimulate the performance of the activity and the debate: role-playing, brainstorming, case study, guided discussion through questions, and so on.  -**Content**: (1) Conceptualization and identification of roles; (2) Consequences, rights, and responsibilities; (3) Coping strategies.  B.- No intervention. | 1/2/3/4.- Cyberbullying: Screening of Peer Harassment | 1.- Changes in Cyberbullying (Post-pretest differences (ANCOVA):  The intervention has shown a significant decrease in Cybervictimization, Cyberperpetration and Cyberaggression-victimization. However no change in Cyber-bystanders has been found.  1.- Victimization F = 13.89 p = 0.000*  2.- Perpetration F = 14.55 p = 0.000*  3.- Observation F = 3.63 p = 0.058  4.- Aggressive-victimi. F = 14.89 p = 0.000* |
| **Gradinger et al.**  **2015**  **Austria** | Reduce Cyberbullying:  1.- Cyberperpetration  2.- Cybervictimization. | QE/2042/No | Students in 5th to 7th grade of secondary schools (10-15 years old). | A.- Experimental group: The ViSC social competence program (1377).  B.- Control group: No intervention (665). | A.- The ViSC social competence program:  -**Duration**: 90 minutes per lesson (over a school year).  -**Format**: Training for teachers and 13 structured lessons  -**Content**: During the program implementation, teachers are trained in (a) how to recognize bullying cases, (b) how to tackle acute bullying cases, and (c) how to implement preventive measures on the school and the class levels. The class  project aims to empower students to take the responsibility for what happens in their class.  B.- No intervention. | 1/2.- Self-reported cyberbullying and cyber victimization were measured with two scales, each containing seven specific items related to different electronic means based on Smith et al. (2008). | 1.- Changes in Cyberperpetration (Mean (SD)):  -Control: t1=0.13 (0.40), t2=0.39 (0.93).  -Experimental: t1=0.20 (0.71), t2=0.33 (0.82).  2.- Changes in Cybervictimization (Mean (SD)):  -Control: t1=0.15 (0.40), t2=0.31 (0.79)  -Experimental: t1=0.21 (0.66), t2=0.27 (0.69).  Mean change of Cyberaggression (Intervention-Control):  Model 1 (traditional bullying) =-0.314; p<0.001  Model 2 (age) =-0.308; p<0.001  Model 3a (boys) =-0.238; p<0.01  Model 3b (girls) =-0.369; p<0.01  Mean change of Cybervictimization (Intervention-Control):  Model 1 (traditional bullying) =-0.199; p<0.01  Model 2 (age) =-0.196; p<0.01  Model 3a (boys) =-0.196; p<0.01  Model 3b (girls) =-0.191; p=0.61 |
| **Guarini et al.**  **2019**  **Italy.** | Reduce Cyberbullying:  1.- Cyberperpetration.  2.- Cybervictimization. | QE pre-post / 898 / No | Students lower secondary schools (6th–8th grades, ages 10-15) | A.- Experimental group: “Relazioni per crescere” - Relationships to Grow (RPC) | A.- RPC program:  **- Duration:** Four in-class activities of 1.5–2 h each during school hours within a two-month period.  **- Format:** In-class activities proposed by teachers including group discussion, work in small groups, and role playing. Dialog, discussion and negotiation among students were encouraged.  **- Content:** The four activities covered the topics: 1) digital literacy, 2) awareness raising and education on cyberbullying, 3) empathy training, and 4) coping skills.  At the end of each activity, students produced materials (posters, slogans, pictures) to synthesize the contents and to keep the main messages for the class in the future. | 1/2.- European Cyberbullying Intervention Project Questionnaire (ECIPQ). | Differences between pre-post intervention scores (multilevel regression analysis):  1.- Cyberperpetration total scores:  - Degree of cyberbullying: t1=1.07 (0.17), t2=1.07 (0.20); t=0.40; β=0.01; p=0.689.  2.- Cybervictimization total scores:  - Degree of cybervictimization: t1=1.13 (0.29), t2=1.14 (0.30); t=0.97; β=0.02; p=0.331.  About 30% of participants, both in pre- and post-intervention, were involved in cyberbullying as bully, victim, or bully-victim. No significant changes in average scores for cyberbullying or cybervictimization were highlighted by multilevel regressions. No gender- or age-related differences were found. |
| **Martínez-Martínez et al.**  **2021**  **Spain.** | 1.- To reduce Cybervictmization | Quasi-experimental pre-post / 330 / 3 months. | Students 7-12 years old (3rd to 6th grade). | A. Experimental group: ANA program. | A.- ANA Program:  **- Duration:** Delivered over two months. **- Format/content:** Eight group sessions in which empathy, assertiveness, communication skills, conflict resolution, and group cohesion were worked on.  Aimed mainly at mobilizing the observers (3 extra sessions for the educational community and two were held for the parents). | 1.- Self-reported Peer Bullying Questionnaire | 1.- Cybervictimization:  The differences were not statistically significant for cybervictimization (t = 0.31, p = 0.756). |
| **Matinez-Vilchis et al.**  **2018**  **Mexico** | Reduce Cyberbullying:  1.- Cyberperpetration  2.- Cybervictimization | QE/82/No | High School students (15-17 years old). | A.- Experimental group: Emotional Competence Program (44).  B.- Control group: No intervention (38). | A.- Emotional Competence Program  -**Duration**: 8 sessions (1 hour per session) over 2 months  -**Format**: 8 different 1-hour online sessions focused on developing basic emotional competences.  -**Content**: activities for the development of the emotional awareness skills, regulation emotional and social competence, which they represent basic skills in development emotional. | 1/2.- Cyberbullying Questionnaire (CBQ). | Differences Control-Intervention (Mann-Whitney U test):  1.- Perpetration:  Pretest: Z=-0.83; p=0.404  Postest: Z=-1.89; p=0.059  2.- Victimization:  Pretest: Z=-1.21; p=0.222  Posttest: Z=-2.96; p=0.003 |
| **Menesini et al.**  **2012**  **Italy** | Reduce Cyberbullying:  1.- Cyberperpetration  2.- Cybervictimization | **Study 1:** QE/386/No  **Study 2:**  QE/375/No | Students from 9^th^ to 13^th^ grade (14-20 years old). | **Study 1:**  A1.- Experimental group: Awareness (126).  A2.- Experimental group: Peer educators (63).  B.- Control group: No intervention (47).  **Study 2:**  A1.- Experimental group: Awareness (189).  A2.- Experimental group: Peer educators (42).  B.- Control group: No intervention (144). | A1.- Awareness group:  -**Duration**: 6 months.  -**Format**: Classes.  -**Content**: Receive only an intervention based on awareness of cyberbullying.  A2.- Peer educators group:  -**Duration**: 6 months.  -**Format**: Online forum and face to face meetings.  -**Content**: Intervention by online educators in the forum controlling the forum posting new threads, answering questions posted by users, moderating discussions. Intervention by face-to-face peer educators: 1) conducting an awareness meeting on bullying and cyberbullying with a school class that had not participated in the previous steps; 2) participating in a meeting with local administrators, police, etc., to ask for specific help making life safer in their city; 3) preparing a TV program about bullying and cyberbullying for a local network.  B.- No intervention. | 1./2- A revised version of the cyberbullying scale described by Menesini, Nocentini, and Calussi (2011). | **Study 1:**  (A1 vs. B; A2 vs. B)  Changes in Cyberbullying (RM ANOVA):  1.- Cyberperpetration:  A significant interaction of time*group (F(4, 28) = 3.408; p<0.05).  A significant interaction of time*group*gender (F(4, 288) = 3.039; p<.05).  2.- Cybervictimization: Nonsignificant.  **Study 2:**  (A1 and A2 vs. B)  Changes in Cyberbullying (RM ANOVA):  1.- Cyberperpetration: Nonsignificant  2.- Cybervictimization:  A significant interaction of time*group (F(2, 75) = 5.706; p<0.05).  (A1 vs. A2)  Changes in Cyberbullying (RM ANOVA):  1.- Cyberperpetration: Nonsignificant.  2.- Cybervictimization: Nonsignificant. |
| **Ortega-Barón et al.**  **2019**  **Spain.** | 1.- Reduce Cyberperpetration.  2.- Reduce Cybervictimization. | RCT/660/No. | Adolescents between 12 and 17 years. | A.- Experimental group: Prev@cib program (434).  B.- Control group: No intervention (236) | A.- Intervention program:  **- Duration:** 10 one-hour sessions with a total duration of 9 months.  **- Format:** 3 modules with 4, 2, and 4 sessions each, administered by teachers and researchers previously trained by one of the investigators in the study.  **- Content:**  Module 1: Information about risk and prevention factors in the bullying and cyberbullying problem.  Session 1. My life is a display window. Session 2. Bullying and cyberbullying. Session 3. Sexting and grooming.  Session 4. Cyber-protection  Module 2: Awareness and sensitization about cyberbullying.  Session 5. Consequences and we are all responsible.  Session 6. What if you were the victim?  Module 3: Involvement in and commitment to prevention and intervention in cyberbullying.  Session 7. What to do when faced with bullying?.  Session 8. (Cyber)helpers.  Session 9. I like myself, I like you.  Session 10. No more bullying.  B. No intervention | 1.- Cyberperpetration was measured through the Scale of Aggression through the Cell phone and Internet (CYB-AGRESS).  2.- Cybervictimization was measured with the Scale of Victimization through the Cell Phone and Internet (CYBVIC). | Between-group effects and repeated-measures ANOVA 2x2 with a between-subjects factor (experimental group and control group) and a within-subjects factor (before and after the program: pre-test and post-test).  1.- Cyberperpetration:  -Experimental group: M (DT) pre-test = 1.21 (0.28), M (DT) post-test = 1.14 (0.32).  -Control group: M (DT) pre-test = 1.24 (0.34), M (DT) post-test = 1.23 (0.41).  -Time effect = 7.39 (p < 0.01), group effect = 7.03 (p < 0.01), interaction effect = 4.67 (p < 0.05), η^2^ = 0.05.  2.- Cybervictimization:  -Experimental group: M (DT) pre-test = 1.27 (0.41), M (DT) post-test = 1.20 (0.32).  -Control group: M (DT) pre-test = 1.28 (0.39), M (DT) post-test = 1.32 (0.47).  -Time effect = 1.16 (p = n.s.), group effect = 6.38 (p < 0.05), interaction effect = 11.63 (p < 0.001), η^2^ = 0.04.  Findings indicated that cyberaggression remained stable in the control group, whereas it decreased in the experimental group. A significant group x time effect was also obtained for cybervictimization F (1, 658) = 11.63; p < 0.001, with a small effect size, η^2^= 0.04. Cybervictimization increased slightly in the control group, whereas it decreased in the experimental group. |
| **Palladino et al.**  **2016**  **Italy** | Reduce Cyberbullying:  1.- Cybervictimization.  2.- Cyberperpetration. | Trial 1: Non-RCT/622/6  Trial 2: Non-RCT/461/6 | Trial 1: Students enrolled in 9^th^ grade (14-18 years old).  Trial 2: Students enrolled in 9th grade (10-16 years old). | Study 1:  A.- Experimental Group: No Trap! Program (451)  B.- Control Group: No intervention (171)  Study 2:  A.- Experimental Group: No Trap! Program (234)  B.- Control Group: No intervention (227) | A.- No Trap! Program:  -**Duration**: A School year.  -**Format**: Training courses, peer-to-peer activities and online forum moderation.  -**Content**: Raising awareness and improving knowledge on issues related to bullying and cyberbullying, training in communication skills, social skills in real and virtual interactions, victims’ and bystanders’ emotions, empathy and adaptive coping strategies-problem solving. Face-to-face peer educator-led activities on victims’ and bystanders’ feelings and emotions and empathy, and on how to cope in bullying and cyberbullying situations. Online peer educators’ intervention as forum moderators.  Several activities focused on improving the social abilities, coping strategies and emotion control. There was one peer educator every 5-7 students helping their classmates through all the intervention /6 months  B.- No intervention. | 1/2.- Florence Cyberbullying-Cybervictimization Scales. | Study 1:  1.- Cybervictimization  A **significant decrease** among the experimental group.  - Control: t1=0.041 (0.07), t2=0.043 (0.11), t3=0.028 (0.06)  -Exp.: t1=0.044 (0.08), t2=0.015 (0.04), t3=0.014 (0.03)  2.- Cyberperpetration:  A **significant decrease** among the experimental group.  - Control: t1=0.031 (0.07), t2=0.047 (0.11), t3=0.016 (0.06)  -Exp.: t1=0.033 (0.07), t2=0.013 (0.04), t3=0.008 (0.02)  Study 2:  1.- Cybervictimization  A **significant decrease** among the experimental group. The intervention had more effectivity reducing cybervictimization among men than women.  - Men: Control: t1=0.053 (0.06), t2=0.056 (0.08)  Exp.: t1=0.057 (0.07), t2=0.029 (0.04)  -Women: Control: t1=0.054 (0.07), t2=0.051 (0.06)  Exp.: 0.052 (0.06), t2=0.055 (0.07)  2.- Cyberperpetration: Nonsignificant. |
| **Pieschl et al.**  **2017**  **Australia** | Reduce Cyberbullying:  1.- Cyberperpetration  2.- Cybervictimization  3.- Cyberbystanding | **Study 1:** QE/143/108/No  **Study 2:** RCT/150/No | **Study1:** Students enrolled in Gymnasium (High Track Secondary School).  **Study 2:** Students enrolled in Gesamtschule (a comprehensive school that accepts students of all ability levels). | **Study 1:**  A.- Experimental Group: Surf-Fair Program (143/108).  **Study 2:**  A.- Experimental Group: Surf-Fair Program (Bystander Unit Alone) (74).  B.- Control Group: No intervention (76). | **Study 1:**  A.- Surf-Fair Program:  -**Duration**: 180 min.  -**Format**: Video presentation.  -**Content**: A fictitious cyberbullying case that ends without solution. In the subsequent modular exercises with an emphasis on group work participants are guided to define the problem and find solution themselves from the perspectives of cyber-victims, cyber-perpetrators, and cyber-bystanders.  **Study 2:**  A.- Surf-Fair Program (Bystander Unit Alone):  -**Duration**: 180 min.  -**Format**: Video presentation.  -**Content**: A photo case story without a solution was used to introduce the different roles of bystanders (reinforcers, assistants, outsiders, and defenders) and stimulate students’ empathy. In small groups, students should resolve the situation from the bystander perspective: They discussed and practiced noticing a situation as cyberbullying, interpreting it as an emergency, feeling responsible for helping, knowing adequate forms of assistance, and implementing those.  B.- No intervention | **Study 1:**  1/2.- This questionnaire measures the frequency of cyber-perpetration and cyber-victimization in the last two months, each with five items (harassment, denigration, impersonation, outing, and exclusion; Willard, 2007).  **Study 2:**  1/2/3.- Questionnaire adapted from the Revised Olweus Bullying Questionnaire (Olweus, 2012). | **Study 1:**  Pre/Post Results across all waves:  1.- Cyber-perpetrators: 2.9%/3.6% (M= 0.01,  SD = 0.10/M= 0.01, SD = 0.10).  2.- Cyber-victims: 5.6%/8.7% (M= 0.03, SD = 0.14/M= 0.03, SD = 0.15).  Neither McNemar tests for the classifications nor mixed ANOVAs for the scale means (cyber-victimization and cyber-perpetration), showed significant main effects of time (within subject: pre vs. post).  **Study 2:**  1.- Pre/Post Results:  1.- Cyber-perpetrators: 19%/15% (M= 0.26,  SD = 0.67/M= 0.17, SD = 0.49)  2.- Cyber-victims: 33%/36% (M= 0.41, SD = 0.69/M= 0.51, SD = 0.87).  3.- Cyber-bystanders: 45%/43% (M= 0.68, SD = 0.98/M= 0.67, SD = 0.99)  Neither Chi-square tests for the classifications nor a mixed MANOVA across the three cyber incident scales with treatment (between-subject: EX vs. CG) and time (within-subject: pre vs. post) as factors showed any significant (univariate or multivariate) main effects or interactions. |
| **Schoeps et al.**  **2018**  **Spain** | Reduce Cyberbullying:  1.- Cyberperpetration  2.- Cybervictimization | QE/360/6 | Students enrolled in 7^th^ to 8^th^ grades (12-15 years old) | A.- Experimental Group: PREDEMA program (168).  B.- Control Group: No intervention. (192). | A.- PREDEMA program:  -**Duration**: Eleven sessions, each of 50 min, which took place over a 3-month period.  -**Format**: The program was implemented in six classes with 25–30 students each.  -**Content**: Sessions 1–6 focused on the most basic emotional abilities, including perceiving, labeling, expressing, using and understanding emotions. Sessions 7–11 targeted emotional regulation and management in different contexts and situations. In addition, complementary issues were discussed, such as personal and global values, responsibility and tolerance, as well as preventing interpersonal conflicts.  B.- No intervention. | 1.- CYB-AG scale  (cyberbullying-aggression).  2.- CYB-VIC scale (cyberbullying-victimization). | Changes in Cyberbullying (Mean (SD)):  1.- Cyberperpetration:  t2: Experimental=11.46 (2.24), Control=13.96 (5.46); p<0.001  t3: Experimental=11.42 (2.05), Control=12.82 (4.86); p=0.01  2.- Cybervictimization:  t2: Experimental=11.88 (2.45), Control=13.92 (5.02); p<0.001 |
| **Solomontos-Kontouri et al.**  **2016**  **Cyprus** | Reduce Cyberbullying:  1.- Cyberperpetration  2.- Cybervictimization | QE/1652/12 months. | Students from 7^th^ and 8^th^ grade (12-15 years old). | A.- Experimental group: The ViSC program (602).  B.- Control group: (1050). | A.- The ViSC Program:  -**Duration**: 90 minutes per lesson (over a school year).  -**Format**: Training for teachers and 6 structured lessons amd a presentation of the ViSC program to the parents.  -**Content**: During the program implementation, teachers are trained in (a) how to recognize bullying cases, (b) how to tackle acute bullying cases, and (c) how to implement preventive measures on the school and the class levels. The class  project aims to empower students to take the responsibility for what happens in their class. A two hours presentation of the ViSC program was offered to the  parents in each school by the researcher, including discussion of how parents  would help.  B.- No intervention. | 1.- Cyberbullying and Cybervictimization scales based on Smith et al. (2008).  2.- Cyberbullying and Cybervictimization scales based on Smith et al. (2008). | Changes in Cyberbullying (Mean (SD)):  1.- Cyberperpetration:  Grade 7  Experimental: t1=0.06 (0.19), t2=0.10 (0.31), t3=0.09 (0.25).  Control: t1=0.08 (0.22), t2=0.12 (0.46), t3=0.13 (0.43).  No intervention effects.  Grade 8  Experimental: t1=0.16 (0.37), t2=0.31 (0.66), t3=0.16 (0.42).  Control: t1=0.12 (0.36), t2=0.13 (0.42), t3=0.14 (0.42).  intervention × time, b = 0.423, p<0.001.  intervention × time^2^, b = −0.195, p<0.001  2.- Cybervictimization:  Grade 7  Experimental: t1=0.12 (0.33), t2=0.14 (0.37), t3=0.12 (0.34).  Control: t1=0.12 (0.34), t2=0.16 (0.46), t3=0.15 (0.44).  intervention × time, b = 0.207, p<0.05  Grade 8  Experimental: t1=0.18 (0.44), t2=0.36 (0.71), t3=0.18 (0.45).  Control: t1=0.18 (0.47), t2=0.16 (0.49), t3=0.15 (0.45).  intervention × time, b = 0.620, p<0.001  intervention × time^2^, b = −0.251, p<0.001 |
| **Sorrentino et al.**  **2018**  **Italy** | Reduce Cyberbullying:  1.- Cyberperpetration.  2._ Cybervictimization. | RCT/759/6 months | Students aged 10-17. | A.- Experimental group: The Tabby Improved Prevention and Intervention Program (TIPIP) (20 classes).  B.- Control group: No intervention (29 classes). | A.- The Tabby Improved Prevention and Intervention Program (TIPIP):  -**Duration**: Teacher training: 4 sessions (12 hours), in-class activities :4 sessions (8 hours).  -**Format**: (i) training activities with teachers, (ii) school conferences with parents; (iii) online materials for students, teachers and parents; and (iv) in-class activities with students.  -**Content**: Cyberbullying and its forms, risk factors, how to recognize it and legal issues.  B.- No intervention. | 1/2.- The Tabby Improved checklist. | Changes in Cyberbullying (Mean (SD)):  1.- Cyberperpetration:  -Experimental: t1=0.29 (0.79), t2=0.21 (0.61).  -Control: t1=0.28 (0.84), t2=0.50 (1.78).  ANOVA: p<0.05.  2.- Cybervictimization:  -Experimental: t1=1.10 (2.11), t2=0.74 (1.27).  -Control: t1=1.12 (1.73), t2=1.31 (2.34).  ANOVA: p<0.001.  Gender-differenciated results:  Boys:  1.- Cyberperpetration:  -Experimental: t1=0.35 (0.79), t2=0.24 (0.65).  -Control: t1=0.47 (1.08), t2=0.93 (2.55).  ANOVA: p<0.05.  2.- Cybervictimization:  -Experimental: t1=1.18 (2.17), t2=0.63 (1.12).  -Control: t1=1.08 (1.75), t2=1.52 (2.92).  ANOVA: p<0.001.  Girls:  Nonsignificant differences |
| **Sullivan et al.**  **2021**  **USA** | Reduce Cyberbullying:  1.- Cyberperpetration.  2._ Cybervictimization | School-level multiple baseline RCT/ 2725/No.  (collected data from students four times each year, during 8 years). | Students from grades 6th, 7th, and 8th. | A.- Experimental group: The Olweus Bullying Prevention Program (OBPP):  B.- Control group: Added to the experimental group in year 3 and 6 of the intervention.  School-level multiple baseline experimental design (total: 8 years). | A.- OBPP program:  -**Duaration**: 3 to 7 years (depending on the year the intervention was implemented).  -**Format:** Individual component, classroom component, school-level component, community level.  -Content: activities including information about cyberbullying and activities aimed at developing empathy, social-problem solving and coping strategies. | 1/2.- Problem Behavior Frequency  Scale–Adolescent Report Form  (collected data from students four times each year) | 1.- Cyberperpetration:  significant intervention effects were found for ratings of relational and cyber aggression, however, only during the 3rd and subsequent years of implementation  (ds = −0.16 and −0.23).  Intervention effects did not differ across sex or grades.  2.- Cyberictimization:  Intervention effects reflecting decreases in  cyber victimization were evident, beginning in the 2nd year of implementation (d = −0.08), and remaining significant in subsequent years (d = −0.10).  Intervention effects did not differ across sex or grades |
| **Williford et al.**  **2013**  **Finland** | Reduce: (1) Cyberperpetration and (2) Cybervictimization by targeting Bystanders | RCT/18412/No | Grades 4-9 (10-16 years old) | A.- Experimental group: The KiVa antibullying program (9914)  B.-Control group: No intervention (8498) | A.- The KiVa antibullying program:  -**Duration**: Elementary curriculum: 20 hr of classroom-based instruction delivered during a school year (two 1-hour lessons per month). Middle school: activities are based on four successive themes that are delivered four times during the school year.  -**Format**: Classroom lessons and individual and group discussions.  -**Content**: a) Universal actions (classroom-based lessons); b) Indicated actions target specific incidents of bullying, including cyber, through adult intervention and peer support for the victimized student.  B.- No intervention | 1/2.- Modified version of the Olweus Bully/Victim Questionnaire (OBVQ). | 1.- Cyberperpetration:  The KiVa program had a significant effect on cyberaggression for students that were one standard deviation below the mean age (OR=1.69, CI95%=1,15-2,48) precisely, the effect of KiVa on cyberbullying was no longer significant when students were 12.87 years of age. KiVa students reported **lower frequencies of cyberbullying than** students in the control group.  No differences between boys and girls.  2.- Cybervictimization:  The KiVa program had a significant effect on **cybervictimization**, (OR= 1.29, CI95%=1.05-1,57), which means that the probability of cybervictimization in students in control group was 1.29 higher than students in the intervention group.  No differences between boys and girls. |
| **Wölfer et al.**  **2013**  **Germany** | 1.- Reduce Cyberperpetration | Randomized Controlled Trial/593/3 | Students enrolled in 7^th^ to 10^th^ grades (12-14 years old) | A1.- Experimental Group: Media Heroes Long-Version (194)  A2.- Experimental Group: Media Heroes Short-Version (104)  B.- Control Group: No intervention (295). | A1.- Media Heroes Long Version  -**Duration**: 10 weeks with a 90-min session per week.  -**Format**: Classroom sessions, group discussions, role playing, peer-to-peer and peer-to-parents mentoring.  -**Content**: 1.- Pros and Cons of New Media; 2.- Definition and Consequences of Cyberbullying; 3.- Feelings and Perspectives; 4.- Participant Roles; 5.-Internet Safety; 6.- Legal Range of Actions; 7.- Parent evening; 8.- Reflection.  A2.- Same format and content in a short version of 1 day with four 90-min sessions.  B.- They did not receive any kind of intervention. | 1.- Self-constructed questionnaire developed within the framework of the current research project (cf. Brighi et al. 2012; for further information, see also Del Rey et al. 2012). | 1.- Changes in Cyberperpetration (Mean (SD)):  Control Group: 0.12 (1.23); Short Intervention: 0.00 (0.68); Long Intervention: -0.19 (0.68).  MANOVA: 5.42; p<0.01. |
